# Integrated mapping of pharmacokinetics and pharmacodynamics in a patient-derived xenograft model of glioblastoma

**DOI:** 10.1038/s41467-018-07334-3

**Published:** 2018-11-21

**Authors:** Elizabeth C. Randall, Kristina B. Emdal, Janice K. Laramy, Minjee Kim, Alison Roos, David Calligaris, Michael S. Regan, Shiv K. Gupta, Ann C. Mladek, Brett L. Carlson, Aaron J. Johnson, Fa-Ke Lu, X. Sunney Xie, Brian A. Joughin, Raven J. Reddy, Sen Peng, Walid M. Abdelmoula, Pamela R. Jackson, Aarti Kolluri, Katherine A. Kellersberger, Jeffrey N. Agar, Douglas A. Lauffenburger, Kristin R. Swanson, Nhan L. Tran, William F. Elmquist, Forest M. White, Jann N. Sarkaria, Nathalie Y. R. Agar

**Affiliations:** 1 Department of Radiology, Brigham and Women’s Hospital, Harvard Medical School, Boston, MA 02115 USA; 2 Department of Biological Engineering, Koch Institute for Integrative Cancer Research, Massachusetts Institute of Technology, 500 Main St, Cambridge, MA 02142 USA; 3 Department of Pharmaceutics, College of Pharmacy, University of Minnesota, Minneapolis, MN 55455 USA; 4 Department of Cancer Biology, Mayo Clinic, 13400 E. Shea Blvd.MCCRB 03-055, Scottsdale, AZ 85259 USA; 5 Department of Neurosurgery, Brigham and Women’s Hospital, Harvard Medical School, Boston, MA 02115 USA; 6 Department of Radiation Oncology, Mayo Clinic, 200 First St SW, Rochester, MN 55902 USA; 7 Department of Immunology, Mayo Clinic, 200 First St SW, Rochester, MN 55902 USA; 8 Department of Chemistry and Chemical Biology, Harvard University, Cambridge, MA 02138 USA; 9 Cancer and Cell Biology Division, Translational Genomics Research Institute, Phoenix, AZ 85004 USA; 10 Mathematical NeuroOncology Lab, Department of Neurosurgery, Mayo Clinic, 5777 E. Mayo Blvd, Phoenix, AZ 85054 USA; 11 Bruker Daltonics, Billerica, MA 01821 USA; 12 Department of Chemistry and Chemical Biology, Northeastern University, 412 TF (140 The Fenway), Boston, MA 02111 USA; 13 Department of Cancer Biology, Dana-Farber Cancer Institute, Harvard Medical School, Boston, MA 02115 USA; 14 Present Address: Department of Biomedical Engineering, Binghamton University, State University of New York, Binghamton, NY 13902 USA

## Abstract

Therapeutic options for the treatment of glioblastoma remain inadequate despite concerted research efforts in drug development. Therapeutic failure can result from poor permeability of the blood-brain barrier, heterogeneous drug distribution, and development of resistance. Elucidation of relationships among such parameters could enable the development of predictive models of drug response in patients and inform drug development. Complementary analyses were applied to a glioblastoma patient-derived xenograft model in order to quantitatively map distribution and resulting cellular response to the EGFR inhibitor erlotinib. Mass spectrometry images of erlotinib were registered to histology and magnetic resonance images in order to correlate drug distribution with tumor characteristics. Phosphoproteomics and immunohistochemistry were used to assess protein signaling in response to drug, and integrated with transcriptional response using mRNA sequencing. This comprehensive dataset provides simultaneous insight into pharmacokinetics and pharmacodynamics and indicates that erlotinib delivery to intracranial tumors is insufficient to inhibit EGFR tyrosine kinase signaling.

## Abstract (web summary)

Despite major drug discovery efforts, the therapeutic options for glioblastoma (GBM) remain inadequate. Here they analyze patient-derived xenograft model of GBM to quantitatively map distribution and cellular response to the EGFR inhibitor erlotinib, and report heterogeneous erlotinib delivery to intracranial tumors to be inadequate to inhibit EGFR signaling.

## Introduction

Glioblastoma (GBM) is the most common and aggressive form of primary parenchymal brain tumor^1,2^. Treatment typically involves surgery with concurrent radiotherapy and chemotherapy; however, prognosis remains poor with a median survival of just 14–16 months^3^. While numerous potential factors contribute to the poor efficacy of otherwise promising GBM therapies, one major but controversial limitation is heterogeneous drug delivery across the blood–brain barrier and the blood–tumor barrier (hereafter jointly referred to as the BBB). The BBB provides both physical and biochemical barriers to drug delivery into normal brain and excludes the majority of oncologic drugs^4^. The BBB is generally considered to be disrupted in GBM, evidenced by accumulation of normally brain-impenetrant gadolinium (Gd) contrast agent in tumor regions on magnetic resonance (MR) images^5–7^. However, the relationship between imaging-detectable contrast levels and concentrations of small molecules is not well characterized. Moreover, numerous image-guided biopsy studies and patterns of failure following gross total resection of all contrast-enhancing tumor demonstrate, categorically, that a significant portion of all GBM invade tissues beyond the contrast-enhanced regions^6,8–12^. Thus, non-uniform disruption of the BBB may influence drug delivery throughout GBM tumors, and the resulting heterogeneous pharmacodynamic (PD) impact on tumor cell survival may be a critical factor limiting the efficacy of many therapies tested in GBM^5,6,13,14^.

A common genetic feature of GBM is overexpression of epidermal growth factor receptor (EGFR)^3,15^. EGFR gene amplification occurs in ~40% of GBM, and almost half of these tumors have additional truncation or point mutations that result in ligand-independent, high-level constitutive signaling^16^. Dysregulated EGFR signaling promotes cell proliferation, migration, invasiveness, and impaired apoptosis^17^. Erlotinib is a first-generation EGFR tyrosine kinase inhibitor, FDA-approved for the treatment of non-small cell lung cancer, that has significant activity in adenocarcinoma with activating mutations in the EGFR kinase domain^18,19^. Based on the frequency of activation and importance of EGFR signaling, there has been sustained interest in evaluating various EGFR inhibitors in GBM. In pharmacokinetic (PK) studies, the measured concentration of erlotinib in patient cerebrospinal fluid (CSF) was similar to efficacious concentrations in pre-clinical studies; however, little is known about how CSF concentrations of erlotinib relate to the concentrations and heterogeneity of erlotinib distribution in human brain and tumor tissues^20^. In pre-clinical models, erlotinib has limited distribution into the normal mouse brain, and—consistent with the concept that hetereogeneous distribution across the BBB in tumors might limit efficacy—only marginal activity was observed in clinical trials testing erlotinib in patients with newly diagnosed or recurrent GBM^21–23^. Erlotinib is known to be a substrate for the major efflux proteins in the BBB, P-glycoprotein (P-gp) and breast cancer resistance protein (BCRP)^24–26^. The active efflux mechanism limiting erlotinib permeability across an intact BBB, as found in invasive regions of glioma, may be in part responsible for the lack of adequate delivery and hence efficacy in GBM. While these clinical studies demonstrated that erlotinib is not a viable therapy for GBM, there are numerous other small molecule EGFR inhibitors in development with variable distribution across the BBB. Thus, the study reported herein provides a ‘failure analysis’ to understand the limitations of erlotinib tumor distribution and to describe a multi-modal platform that can be used to evaluate and nominate the most promising drugs for evaluation in clinical testing for GBM.

The successful evaluation of new treatments for GBM requires better understanding of the influence of a number of physiologic and pharmacologic parameters. Here we present a novel, multimodal approach to probe these parameters in a GBM patient-derived xenograft (PDX) model in response to erlotinib. Briefly, MR imaging T2-weighted (T2W) hyperintense and T1-weighted Gd contrast-enhanced (T1Gd) image regions allowed approximate delineation of tumor. Ex vivo tissue sections were analyzed by matrix-assisted laser desorption/ionization mass spectrometry imaging (MALDI MSI) to analyze drug and drug metabolite distribution, and ions identified as metabolic biomarkers of the tumor allowed accurate delineation of tumor regions. Imaging of serial sections by stimulated Raman scattering (SRS) microscopy produced high resolution images of the distribution of proteins, lipids and heme, enabling differences in brain and tumor tissue micro-architecture to be assessed. MS images were coregistered with the MR images to provide a comprehensive understanding of drug distribution across various imaging parameters. The MALDI MSI evaluation of drug levels was paired with laser capture microdissection (LCM) and subsequent analysis of transcriptional response in regions of heterogeneous drug delivery. A comparative study between intracranial (IC) and flank PDX was included in the experimental design to evaluate differences in drug delivery and the effect of the BBB on treatment response at the transcriptional and protein signaling levels. Collectively, this multimodality analysis established a spatially resolved PK-PD model for erlotinib and demonstrates that this platform is a robust and comprehensive assessment tool, that could be applied to essentially any novel therapeutic strategy targeting GBM or other tumor where differential drug distribution may be an important factor governing therapeutic response.

## Results

### Multimodality analysis for integrated PK and PD

**Fig. 1:**
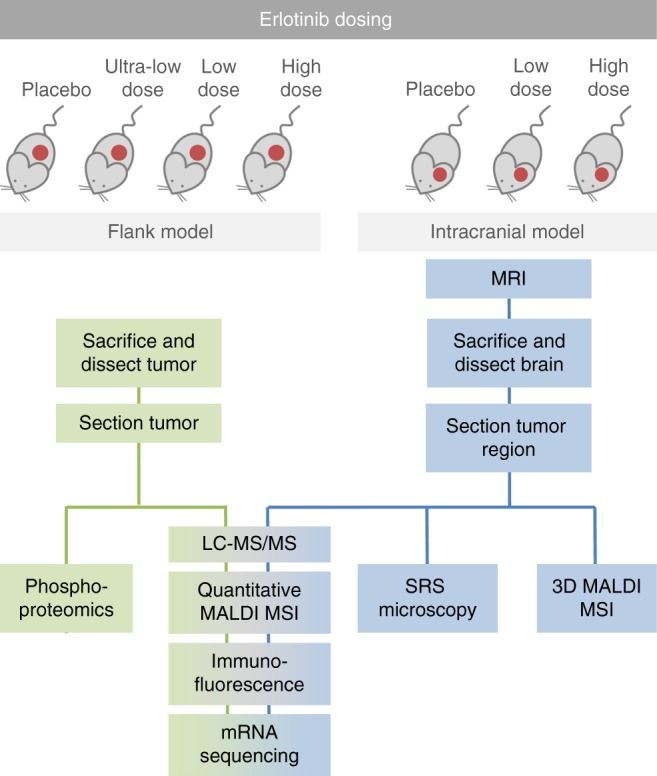
Schematic of multimodal analysis workflow for investigation into drug delivery and response. Two groups of mice were injected with patient-derived GBM12 tumor cells to establish xenografts either in the flank or brain. Mice in both groups were treated with either placebo, ultra-low dose (5 mg kg^−1^), low dose (33 mg kg^−1^), or high dose (100 mg kg^−1^) erlotinib. Tissue from mice in each group was analyzed according to the workflow shown in the schematic

This study was designed to investigate the distribution of and cellular response to erlotinib, an EGFR inhibitor, in both flank and IC GBM PDX models and is outlined in Fig. 1. Mice in both flank and IC groups were dosed with placebo, low (33 mg kg^−1^) or high (100 mg kg^−1^) dose erlotinib, and an additional flank group was dosed at ultra-low dose (5 mg kg^−1^). Drug levels in plasma, brain, and flank tumors were determined by liquid chromatography–tandem mass spectrometry (LC–MS/MS). Tissue samples were then serially sectioned and processed for multimodality imaging. Detailed analyses (MRI, 3D MALDI MSI, histology, SRS microscopy, and mRNA sequencing) were performed on tissue from one high dosed animal with IC PDX to provide comparative data for integrated PK and PD. Further analyses were performed on tissue from animals with flank and IC PDX under different doses of erlotinib to investigate dose-dependent response and differences in drug delivery and distribution.

### Heterogeneous drug distribution observed by 3D MALDI MSI

**Fig. 2:**
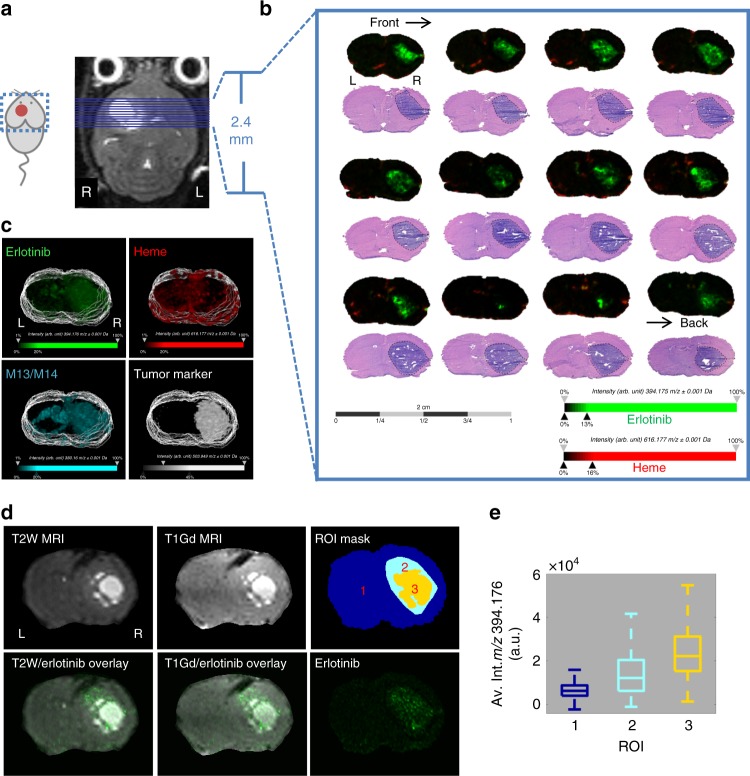
Multimodal 3D imaging with MALDI MSI, MRI and H&E, letters ‘L’ and ‘R’ denote left and right sides of the mouse brain. **a** contrast-enhanced T1-weighted MR image of mouse with established intracranial tumor. **b** MALDI MS imaging of serial coronal sections of mouse brain (subset from tumor core only displayed, see Supplementary Figure 2 for full set of images) with established GBM tumor after treatment with 100 mg kg^−1^ erlotinib (2 h post-dose) with corresponding H&E staining of adjacent sections (ion images of erlotinib (green, *m/z* 394.176 ± 0.001) and heme (red, *m/z* 616.177 ± 0.001) overlaid, **c** 3D reconstructions of MALDI ion images collected at 160 μm intervals and imaged at 100 μm spatial resolution, Erlotinib (green, *m/z* 394.176 ± 0.001), heme (red, *m/z* 616.177 ± 0.001), the active metabolite of erlotinib (M13/M14) (cyan, *m/z* 380.160 ± 0.001), and a tumor biomarker (white, *m/z* 503.949 ± 0.001), **d** automatic multimodal non-rigid alignment of erlotinib ion image with T2W and T1Gd MR images of one example section/plane, and regions of interest (ROI) mask image for normal brain (1), tumor associated drug exclusive of T1Gd hyperintensity (2), and T1Gd hyper intensity (3) were generated from the registered T1Gd MR and erlotinib ion images, and **e** boxplot of erlotinib intensity detected in each ROI, where the center line represents the median, the upper and lower bounds of the box represent the inter-quartile range and the whiskers represent ±1 standard deviation of the mean

Brain tissue from an animal bearing an IC tumor and dosed with 100 mg kg^−1^ erlotinib was harvested and sectioned for multimodal imaging. 3D MALDI MSI acquired on a 9.4 T Fourier transform ion cyclotron resonance (FT-ICR) MS was used to measure the distribution of drug and metabolite in relation to endogenous biomolecules. Coronal sections of the tumor region were taken every 160 µm, as outlined in Fig. 2a, b. Serial sections were stained with H&E for comparison. Images of ions with *m/z* 394.1760 (Δppm = 0.25) (Fig. 2b and Supplementary Figure 1) indicate that the parent drug (erlotinib) is distributed with higher intensity in the highly cellular tumor regions compared with normal brain parenchyma. The drug is not distributed homogeneously through tumor, but instead appears with varied intensity in the *x*, *y*, and *z* dimensions. Images of ions with *m/z* 616.1770 (Δppm = 0.49) correspond to heme (Fig. 2b); images of heme have previously been shown to mark the vasculature^27^. Overlaid images of erlotinib and heme show low colocalization between the blood vessels and the drug over the full tissue sections including both tumor and healthy brain (Fig. 2b, c and Supplementary Table 1). Both the parent drug and one of its metabolites (M13/M14)^27,28^ were detected with higher intensity in the tumor compared with non-tumor tissue, suggesting that the BBB is disrupted to a greater degree within tumor regions. The parent drug was detected with ~10-fold higher intensity than the metabolite (Supplementary Figure 2).

3D MALDI MS ion images are shown for erlotinib, heme, the active metabolite of erlotinib (M13/M14) and unidentified ion with *m/z* 503.949, which was found to map to the tumor region by comparison with H&E (see Fig. 2c). The use of internal MALDI MSI ‘biomarkers’ to map different tissue features can provide a more accurate comparison than H&E or other staining techniques because serial sections undergo separate preparation and are subject to different distortions. Interestingly the parent and metabolite demonstrated different distributions, with the metabolite distributed more broadly, than the parent drug, and a relatively high concentration detected in the lateral ventricles (Fig. 2c). Co-localization between different ions was measured by calculating Pearson’s correlation coefficient between pairs of ion images (for the whole three-dimensional dataset), see Supplementary Table 1. Correlation values range between 0 (indicating no co-localization/inverse distribution) and 1 (indicating perfect co-localization). The highest co-localization was observed between the parent drug and drug metabolite, and both the parent drug and metabolite showed similar co-localization with the tumor biomarker, indicating similar specificity for the target region.

### Comparative analysis of MRI and MALDI MSI

MR images and 3D MALDI images from the same animal were co-registered to establish the relationships among BBB disruption, drug distribution, and tumor microenvironment. To assess the distribution of drug in relation to histology and MR imaging (T1Gd and T2W MR images), a non-rigid registration was applied to a reduced MALDI MSI dataset, and erlotinib was mapped to the aligned coordinates. Overlaid images between drug and T2W MR images can be seen in Fig. 2d. Registration between the drug ion image and H&E histology reveals the extent of heterogeneity in drug distribution within the tumor region (see Supplementary Figure  3). Regions of high contrast were similar in T1Gd and T2W MR images, Fig. 2d. Drug signal intensity was highest within the regions of high contrast in MR images, as measured using a mask image of three different regions of interest (ROIs) segmented from T1Gd MR and erlotinib images (Fig. 2d, e). The low intensity of erlotinib at the invasive margins of the tumor (beyond contrast enhanced region of MR images) suggests that significant fractions of the tumor cells are underexposed (Fig. 2d). Taken together, these data indicate that erlotinib drug levels were elevated but highly variable within the tumor.

### High-resolution imaging with SRS microscopy

**Fig. 3:**
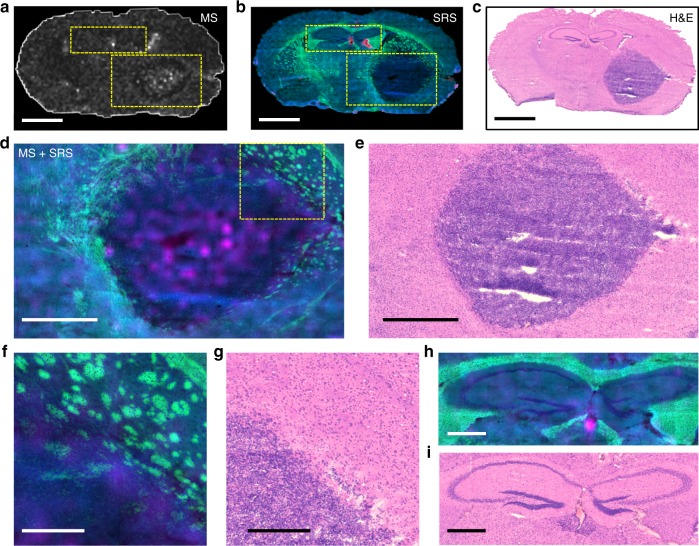
Correlated MALDI MS imaging of the drug (erlotinib) distribution and SRS imaging of the brain tissue morphology. **a** MALDI MS ion imaging shows the distribution of Erlotinib across the entire brain tissue section. **b** SRS imaging shows the chemical constituents (lipid in green; protein in blue; and heme in red) of the entire serial brain tissue section. **c** H&E image of another serial brain tissue section corresponding to **a** and **b. d** Enlarged overlaid view of areas marked by the large rectangle in **a** and **b** shows distribution of the drug (erlotinib in pink) and tissue morphology (lipid and protein in green and blue, respectively) of the tumor area, **e** H&E image corresponding to **d**. **f** Enlarged overlaid images of the tumor margin area (marked by rectangle in **d**) shows cancer cell invasion into the normal fatty brain structure. **g** H&E image corresponding to **f**. **h** Enlarged overlaid images of the brain structure (marked by smaller rectangle on **a** and **b** featuring the difference between gray (protein rich—blue) and white (lipid rich—green) matter. High concentration of erlotinib was found in the third ventricle **h**, with corresponding H&E image shown in **i**. Scalebars represent lengths as follows: **a**–**c** = 1 mm, **d** and **e** = 500 µm, **f**–**i** = 200 µm

SRS microscopy was used to provide high spatial resolution images of chemical constituents of the brain and tumor microenvironments. SRS images of protein, lipid and heme (absorption) in serial sections of brain used for 3D MALDI were acquired (see Fig. 3). Multicolor SRS images revealed cellular morphologies, and demonstrated relative levels of different chemical classes in cells and tissue (Fig. 3b): protein (blue), lipid (green), and heme (red). Higher levels of protein were detected in the tumor core due to the highly-packed cancer cell bodies (Fig. 3b–e). At the brain/tumor boundary, compression or reorganization of the myelinated axon fibers at the tumor margin and cancer cells invading into the normal brain tissue were observed (Fig. 3f–g). In the normal brain structure, white matter (high lipid content, in green) and gray matter (high protein content, in blue) were distinguished due to the lipid/protein concentration difference (Fig. 3h–i). Corresponding H&E images (Fig. 3c–i) of serial sections demonstrated the ability of SRS imaging to provide detailed spatial and chemical information that could not be provided by standard histology.

Overlaid, co-registered images of erlotinib (MALDI-MSI) and SRS images provided a means to investigate drug distribution and its correlation with the brain tissue structure, morphology, and chemical components (Fig. 3d–h). Images demonstrated that the protein rich tumor core contained higher concentrations of the drug. At the tumor edge (Fig. 3f) an inverse relationship with a higher lipid content (green) and lower drug content (pink) was observed. A small, highly localized region of high drug concentration (pink) was detected in the third ventricle (Fig. 3h). Together, such initial observations suggest that erlotinib can enter the tumor core but has limited diffusion across the intact BBB within regions of normal brain, at the infiltrative edge of the tumor and at the interface between tumor and white matter.

### Drug delivery is limited at the invasive tumor edge

Quantitative MALDI MSI was performed to measure absolute concentration per pixel in brain and flank tumors under the low and high doses of erlotinib. A tissue mimetic model^29^ containing known quantities of erlotinib was analyzed by MALDI MSI and a calibration curve was produced (Supplementary Figure 4). The calculated drug concentration in the normal brain, ROI (tumor regions defined by bisecting *k*-means clustering of MALDI MSI data) and flank tumors measured by MALDI MSI were similar to those measured using an analytical LC–MS/MS assay in normal and tumor-bearing (ipsilateral to tumor site) tissue, thereby validating the use of MALDI MSI for erlotinib quantification (Table 1). Quantitative MALDI MSI, was only performed on one animal in each treatment group, but agreement with LC–MS/MS measurements (*n* = 3 for flank, *n* = 2 for IC) suggests the animals analyzed by MALDI MSI were representative of the group and also provides a measure of variation between animals. The brain-to-plasma ratios of erlotinib obtained in this study were similar to those previously measured in a rat glioma model.^26^ Average concentration of erlotinib (measured by MALDI MSI) in IC PDX dosed at 33 and 100 mg kg^−1^ were found to be similar (2354.2 and 2182.3 ng g^−1^, respectively), with slightly higher concentration detected in the tumor dosed at 33 mg kg^−1^ (Table 1). This could be due to a relatively well vascularized tumor situated closer to the brain surface in the 33 mg kg^−1^ dosed animal (Supplementary Figure 5). Further segmentation of the IC tumor into the core and invading edge (Supplementary Figure 6) reveals ~two-fold decrease in drug concentration at the edge relative to the tumor core (at 100 mg kg^−1^ dose). No difference in drug concentration was observed in flank tumors between the core and edge. The concentration of erlotinib was similar in IC tumors dosed at 33 and 100 mg kg^−1^, the average concentrations of erlotinib in flank tumor was dose-dependent at 2426.8 and 8211.6 ng g^−1^, for 33 and 100 mg kg^−1^, respectively. These concentration values were next used to correlate dose-related response detected in further analysis of IC and flank tumors.

**Table 1:** Comparison of tissue-to-plasma ratio and concentrations of erlotinib measured by MALDI MSI and LC-MS/MS in IC and flank tumors and normal brain, whole brain and tumor bearing brain in animals treated with a low dose of erlotinib (33 mg kg^−1^) and a high dose (100 mg kg^−1^)

Tumor location/region	Low dose (MALDI-MSI) (ng g^−1^)	High dose (MALDI-MSI) (ng g^−1^)	Low dose (LC–MS/MS) (ng g^−1^)	High dose (LC–MS/MS) (ng g^−1^)
Flank tumor	2426.8	8211.6	2476.6 ± 1339.9	6267.2 ± 2544.7
*Brain tissue*				
Normal brain^a^	619.3	1082.9	608.4 ± 145.2	3218.8
Tumor only	2354.2	2182.3	NA	NA
Tumor core	2411.5	2787.1	NA	NA
Tumor edge	2011.2	1446.5	NA	NA
Whole brain^b^	NA^d^	NA	471.2 ± 282.6	1337.6 ± 400.4
Tumor-bearing brain^c^	NA	NA	719.7 ± 355.0	3677.2
*Tissue-to-plasma ratio*				
Flank tumor	NA	NA	0.54 ± 0.11	0.63 ± 0.18
Normal brain	NA	NA	0.11 ± 0.04	0.18 ± 0.08
Whole brain	NA	NA	0.10 ± 0.02	0.14 ± 0.02
Tumor bearing brain	NA	NA	0.14 ± 0.08	0.21 ± 0.09

Data are presented as mean (over ROI in MALDI MSI data, *n* = 1) or mean ± standard deviation (intra-animal LC–MS/MS measurement, *n* = 2 for brain, *n* = 3 for flank)

^a^Non-tumor bearing brain (left hemisphere; contralateral to the injection site) obtained from GBM12 intracranial (orthotopic) xenografts

^b^Brain tissue obtained from mouse bearing flank GBM12 xenografts

^c^Right hemisphere (ipsilateral to the injection site) obtained from GBM12 intracranial xenografts

^d^NA—not analyzed

Correlation coefficients between drug and heme were calculated between different image regions (Supplementary Table 2), providing an indication of the availability of the drug (to tumor cells), alongside absolute concentration of drug in the region. Broadly, higher correlation between drug and heme was observed in IC tumors compared to flank, suggesting that the drug is less available in the IC tumor, regardless of the relative concentration. Higher correlation between drug and heme was also observed in the IC tumor edge compared with corresponding tumor core.

### Erlotinib activity is reduced in IC tumors relative to flank

**Fig. 4:**
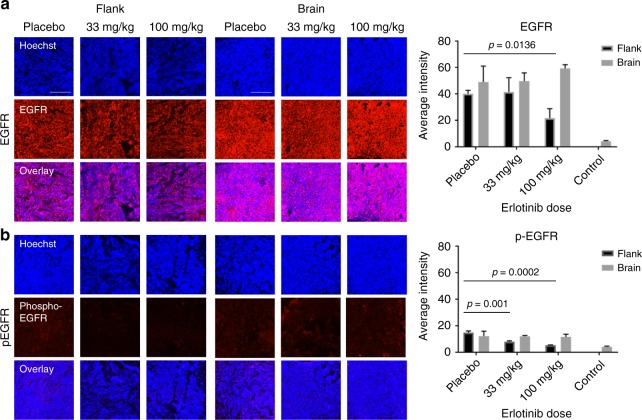
Tissue Immunofluorescence imaging of total EGFR and phosphorylated-EGFR (p-EGFR) in brain and flank tumors. **a** Images showing total EGFR in brain and flank tumors (red) overlaid with Hoechst (blue), treated with placebo, low (33 mg kg^−1^) and high (100 mg kg^−1^) dose erlotinib, and graphs (on right) showing quantitative analysis EGFR levels over whole tumor region in each treatment group. **b** Images showing p-EGFR in brain and flank tumors (red) overlaid with Hoechst (blue), with placebo, low (33 mg kg^−1^) and high (100 mg kg^−1^) dose erlotinib and graphs (on right) showing average (mean) intensity of p- EGFR levels in each treatment group. (scalebars on images represent 250 µm, error bars represent 1 standard deviation, *n* = 2 for brain, *n* = 3 for flank)

Tissue immunofluorescence (IF) was used to visualize and relatively quantify EGFR and phosphorylated-EGFR (p-EGFR) in a further set of IC and flank tumors under placebo, low and high dose of erlotinib. Hoechst images were used to define tumor ROIs based on regions of high cellularity; the tumor was segmented from normal brain (Supplementary Figure 7), and flank tumors were segmented from background. Average intensity for total EGFR and p-EGFR was calculated (see Fig. 4). Total EGFR was detected with higher relative intensity in IC tumor vs. normal brain tissue (Supplementary Figure 7). There was no significant difference in total EGFR in IC tumor regions between placebo and treatment with either low or high dose of erlotinib (*t* = 1.183, degrees of freedom (df) = 2, *p* = 0.358). In flank tumors however, there was a decrease in the amount of total EGFR after treatment with high dose erlotinib, relative to placebo (*t* = 4.213, df = 4, *p* = 0.0136). A similar relationship between flank and IC tumors was observed in relative levels of p-EGFR; on average there was no change in p-EGFR in IC tumors treated with 100 mg kg^−1^ erlotinib, relative to placebo (*t* = 0.162, df = 2 *p* = 0.8859), however, heterogeneity in signal intensity was observed in all IC tumors, which could mask localized regions of response, see Supplementary Figure 8. A decrease was observed in p-EGFR in flank tumors after treatment with low (*t* = 8.562, df = 4, *p* = 0.001) and high (*t* = 13.069, df = 4, *p* = 0.0002) dose erlotinib, relative to placebo. This result is consistent with a dose–response relationship where a lower availability of erlotinib in IC tumors vs. flank results in insufficient suppression of EGFR activity.

### A low dose of erlotinib inhibits EGFR in flank

**Fig. 5:**
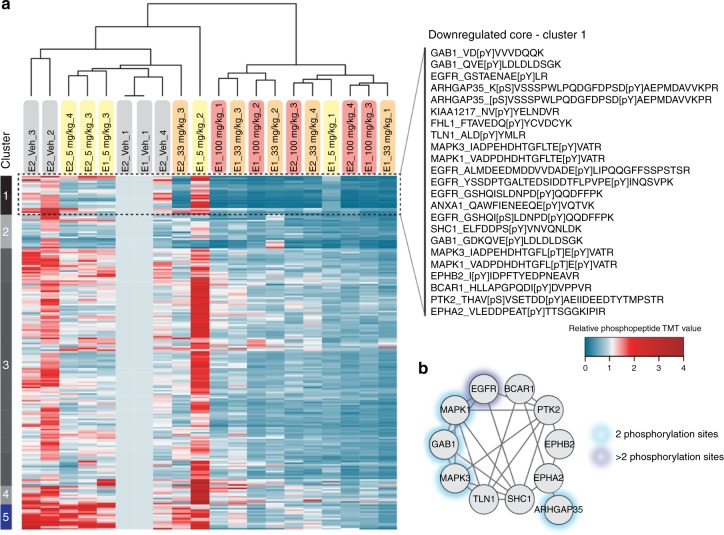
In vivo phosphorylation changes in erlotinib-treated flank tumors reveal inhibition of EGFR signaling. **a** Hierarchical clustering (Euclidian distance) of phosphopeptide changes for GBM12 flank tumors treated with 5, 33, and 100 mg kg^−1^ erlotinib (*n* = 4) and displayed relative to a vehicle-treated flank tumor measured in both of the analyzed TMT-10-plex MS runs (E1 and E2), **b** Phosphoprotein interaction network based on STRING for members of cluster 1 in **a** and represented by their gene name

Phosphoproteomic analysis was performed on tissue samples from flank tumors to assess phosphorylation changes in signaling pathways in response to different doses of erlotinib. Since phosphoproteomic analyses require considerable tissue quantities and are sensitive to post-resection ischemia, our studies were limited to larger and more quickly resectable flank tumors^30^. Phosphoproteomic analysis identified 210 unique phosphotyrosine-containing peptides across the four treatment groups (vehicle, 5, 33 , 100 mg kg^−1^). Quantifying these phosphorylation sites across the tumor treatment conditions revealed inter-tumor heterogeneity in the pre-treatment and ultra-low dose erlotinib (5 mg kg^−1^) tumors (see Fig. 5). Upon treatment with erlotinib (33  and 100 mg kg^−1^), tumor heterogeneity decreased as a consequence of similar responses to signaling inhibition. For these higher dose conditions, we observed a pronounced inhibition of the target (EGFR), proximal adaptor proteins (Gab1, Shc1), and downstream signaling (MAPK1, MAPK3) suggesting that 33 mg kg^−1^ is an adequate dose to inhibit EGFR signaling in flank tumors. Despite the decrease in heterogeneity on treatment, individual tumors treated with 33 or 100 mg kg^−1^ erlotinib demonstrated increased phosphorylation of selected nodes, potentially indicative of resistance pathways beginning to emerge in these tumors (Supplementary Dataset 1). Although no consensus resistance pathways were detected in all of the treated tumors, p130Cas (BCAR1) and PI3K (p110/p85) tyrosine phosphorylation were increased in several tumors in response to 5 and 33 mg kg^−1^. To assess the functional relevance of these increased phosphorylation sites, additional time points or co-treatment, for instance with PI3K inhibition, would be required.

### Dose-dependent transcriptional response in flank and IC

**Fig. 6:**
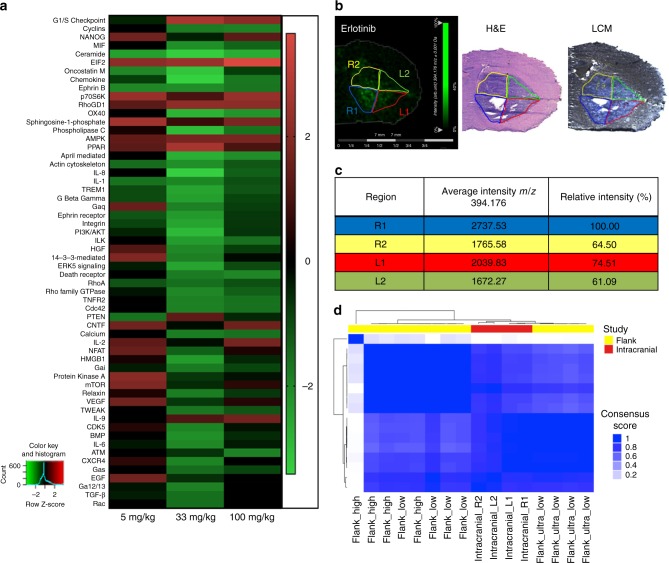
mRNA sequencing of flank and intracranial (IC) tumor tissues from animals dosed with erlotinib showing dose-dependent pathway regulation. **a** Summary of pathways regulated by different doses of erlotinib in flank tumors (*n* = 5), *z*-Scores of significant pathways relative to placebo are presented. **b** MALDI MS images of erlotinib in brain tissue with IC tumor and regions of interest (R1, R2, L1 and L2) used for laser capture microdissection (LCM) and subsequent mRNA sequencing, adjacent section with H&E staining and optical image of adjacent section used for LCM. **c** Average (mean) relative intensity of erlotinib (*m/z* 394.176) detected in each ROI of MALDI MS ion image. **d** Clustermap of transcripts from flank and IC tumors by mRNA sequencing

Transcriptional response to variable erlotinib concentration in flank tumors was evaluated by mRNA sequencing. We selected differentially expressed genes (*q* < .05) from a group comparison of the different treatment groups (5 mg kg^−1^ vs. placebo, 33 mg kg^−1^ vs. placebo group, and 100 mg kg^−1^ vs. placebo group) and utilized Ingenuity comparison analysis to identify canonical pathways activated and repressed (*Z* > 1.5 and *q* < .05). We observed unique and shared pathway signatures among treatment groups (Fig. 6a). All erlotinib-treated groups had activated eukaryotic initiation factor 2 (EIF2), peroxisome proliferator-activated receptor (PPAR), AMP-activated protein kinase (AMPK) signaling and decreased actin cytoskeleton and Ephrin B signaling compared to placebo treated tumors. Additionally, Rho GTPase activity signature was decreased and RhoGDI signaling was increased. Specifically, CDC42, RhoA, and Rac signaling were decreased in erlotinib-treated tumors compared to placebo. We observed that treatment with 5 mg kg^−1^ erlotinib was insufficient to inactivate EGF, hepatocyte growth factor (HGF), 14-3-3, protein kinase A, integrin, and phospholipase C signaling pathways. Compared to flank tumors with low dose erlotinib, higher erlotinib doses activated the G1/S cell cycle checkpoint and interleukin 9 (IL9) pathway, and repressed EGF, cyclin, and death receptor signaling pathways.

To correlate drug exposure/response findings from the flank tumors to IC tumor response, LCM was used to dissect IC tumor tissue (dosed with 100 mg kg^−1^ erlotinib) into four separate quadrants (Fig. 6b). MALDI MS ion images of the drug in a serial tissue section was used to determine the relative concentration of drug in each quadrant dissected (Fig. 6b, c). Relative drug concentration per quadrant varied from 100% in quadrant R1 to 75% in quadrant L1 and 64.5% and 61.09% in quadrants R2 and L2, respectively. Cluster analysis of transcripts from each quadrant show dose-related response highlighting heterogeneity in IC drug distribution and associated response. For this analysis, all genes were taken into account, limiting the differential gene expression. The overall transcripts from IC tumor dosed with 100 mg kg^−1^ erlotinib clustered between transcripts from flank tumors dosed with ultra-low and low dose erlotinib. The centre portions of IC tumors dosed with 100 mg kg^−1^ receive similar concentration of drug as flank tumors dosed with 33 mg kg^−1^ and the edge portions of IC tumors receive ~two-fold less, as determined by MALDI MSI quantification. Transcripts from IC tumors are therefore consistent with the dose–response relationship observed in flank tumors, Fig. 6d.

### Integration of phosphoproteomic and mRNA sequencing data

**Fig. 7:**
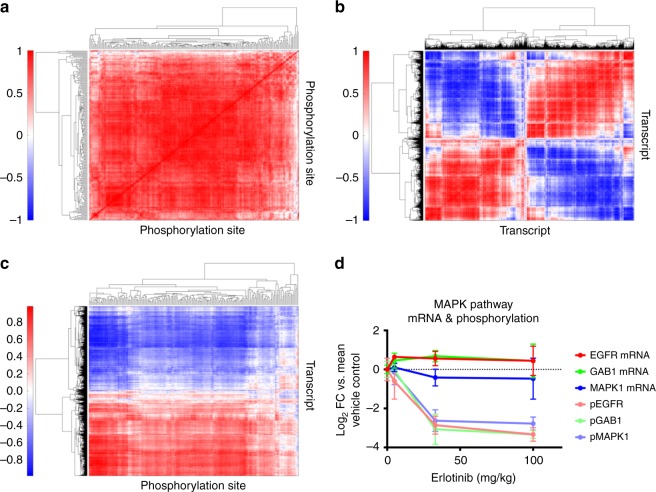
Integrative analysis of phosphoproteomic and mRNASeq data. **a** Hierarchical clustering of correlation matrix for phosphoproteomic data reveals that most sites are correlated. Sub-clusters highlight known interactions as potentially novel signaling network. **b** Clustering of mRNA correlation matrix highlights the bi-modal response to Erlotinib, with most transcripts either decreasing or increasing on Erlotinib treatment and thus either positively or negatively correlated with other transcripts. **c** Clustering of phosphoproteomic and mRNASeq correlation matrix. Phosphorylation sites tend to decrease on Erlotinib treatement and are therefore positively correlated with approximately half of the transcripts and negatively correlated with the other half of the transcripts. Sub-clusters of phosphorylation sites indicate well-established signaling networks. **d** Line graphs showing analysis of correlation between phosphorylation sites and their transcripts indicating that activation/inhibition of the kinase may be regulating the site. Error bars represent the standard deviation

We have previously shown that correlating phosphoproteomic data across different tumors enabled the identification of activated signaling networks^31^; here we applied this same approach to identify co-regulated networks of phosphorylation sites across tumors and treatment conditions. Hierarchical clustering analysis of the correlation matrix of the 210 tyrosine phosphorylation sites across tumors yielded a few clusters (Fig. 7a, Supplementary Dataset 1), including a large, highly correlated cluster for most of the phosphorylation sites in the middle of the clustergram. This large cluster demonstrates that, despite the inter-tumor heterogeneity, most of the sites were co-regulated across tumors and treatment conditions. Smaller sub-clusters were also evident within the data, including a cluster of highly co-regulated sites comprised of four EGFR phosphopeptides, phosphorylation sites on the proximal adaptor proteins and direct EGFR substrates SHC and GAB1, the activation loop phosphorylation sites on the ERK1 and ERK2 mitogen-activated kinases (MAPK3 and MAPK1), and a site on ARHGAP35 (p190RhoA). The ERK MAPKs are known to be directly downstream of EGFR/SHC/GAB1 activation, and p190RhoA is involved in the EGFR pathway as well^32^. Importantly, this sub-cluster demonstrates the ability of the analysis to extract well-characterized signaling networks, while other sub-clusters highlight both known and potentially novel signaling interactions. Using a similar clustering/correlation-based approach to interrogate the quantitative mRNAseq data led to the identification of two large, anti-correlated clusters (Fig. 7b) comprised of many smaller sub-clusters. These large clusters were predominantly defined by sites that increase or decrease upon EGFR inhibition, and are anti-correlated due to their differential response to erlotinib.

Quantitative phosphoproteomic and mRNASeq results were correlated across tumors to attempt to identify co-regulated networks of signaling and transcripts (Fig. 7c, Supplementary Dataset 2–3). This analysis was dominated by the direction of the fold-change on treatment with inhibitor, with most of the phosphorylation sites and approximately half of the transcripts decreasing on treatment with erlotinib; these sites and transcripts were therefore found to correlate, while the other half of the mRNA data was anti-correlated with both. Interestingly, this analysis also extracted well-characterized signaling networks, presumably due to their coordinated response to perturbation. For instance, a cluster of phosphorylation sites with some of the strongest correlation/anti-correlation with mRNA includes six EGFR peptides, three GAB1 peptides, SHC, the ERK 1/2 MAPK activation loop phosphorylation sites, ARHGAP35, and Annexin A1, all well characterized members of the EGFR signaling network^33^. As shown in Fig. 7d, phosphorylation sites on given proteins were not necessarily correlated with their respective transcripts; for instance the phosphorylation of EGFR, the proximal adaptor protein GAB1, and the downstream MAPK1 proteins all decrease significantly with increasing erlotinib concentration, while their respective transcript levels either increase (EGFR, GAB1) or decrease slightly (MAPK1). As this example demonstrates, acquisition of mRNA and protein phosphorylation data on the same tumors enables the determination of whether the change in phosphorylation was likely due to altered signaling, rather than altered protein expression due to transcription and translation.

## Discussion

Differences in the overall average drug concentration in IC compared to flank tumors do not sufficiently account for the lack of efficacy of erlotinib in GBM. Overall average drug concentrations of erlotinib in IC tumors at low and high doses were similar to flank tumors at the low dose. Tissue IF reveals that decreased levels of p-EGFR were associated with both low and high dose of erlotinib relative to placebo in flank tumors, but not in IC tumors with similar erlotinib concentration. Considering these results together it would appear that the same concentration of drug in IC and flank tumors did not have the same effect on EGFR. Closer inspection of MALDI MSI data, however, revealed that different concentrations of erlotinib were detected in different regions of the IC tumor; with a higher concentration (~two-fold higher) measured in the core of the tumor vs. the edge. Furthermore, co-localization between heme and erlotinib images provided an indication of the availabillty of the drug, i.e., whether the drug had left the blood vessels and entered brain/tumor tissue. Higher correlation between drug and heme was observed in IC tumors compared to flank, which suggested that the drug was less available in IC tumors. The highest correlation between drug and heme was observed in the tumor edge, further suggesting that the drug was least available to the infiltrating portion of the tumor. So, while the tumor core received quantities of drug that had the desired therapeutic effect in flank, the tumor edge did not. The combination of lower concentration of drug and low availability at the tumor edge could be a mechanism of failure of erlotinib.

Registration of SRS microscopy images and MALDI ion images of erlotinib enabled the distribution of drug to be related to chemical composition of tissue. Erlotinib showed an almost inverse relationship with regions of high lipid concentration. The region of highest intensity in the drug image corresponded with the protein-rich tumor core. Lower relative concentrations of drug were detected in and at the interface with the lipid-rich regions of white matter. The combination of these two imaging modalities demonstrates how drug distribution relates to global chemical content of various tissues and further demonstrates the limited and heterogeneous nature of drug distribution in the tumor. Differences in non-specific binding in different tumor regions can lead to varying concentrations of free (active) drug due to region-specific changes in the fraction unbound. Differences in the chemical composition of the tissue, such as the tumor core, peripheral tumor rim, normal brain, and even a tumor in the flank, may lead to differences in free drug concentrations, and hence be a factor in determining efficacy^34^.

Registration of T1Gd and T2W MR images with MALDI ion images of erlotinib demonstrated that drug distribution overlapped with the contrast-enhanced portion of the tumor and the area of high drug intensity was larger than the contrast-enhanced portion. This suggested that Gd contrast agent and erlotinib have different distributions, as would be expected of molecules with different physico-chemical properties and PK, but which is mostly disregarded when using contrast enhancement MR to indirectly evaluate drug distribution. Further, although there were generally increased levels of drug within the MR image abnormality, the drug levels were highly variable. This heterogeneity in drug delivery to both the highly cellular tumor regions and the diffusely invaded regions of the brain could provide an opportunity for tumor cells to escape exposure supporting the continued tumor progression seen experimentally^14^.

Activation or inhibition of protein tyrosine phosphorylation signaling networks should lead to consequent changes in transcriptional networks, which may themselves feed back to alter phospho-signaling; together, these regulatory networks control many of the cellular responses to perturbations, including treatment with kinase inhibitors. Integrated phosphoproteomics and mRNA sequencing data revealed that inhibition of protein signaling pathways did not necessarily correlate with respective transcription levels. Changes in the phosphorylation of EGFR, GAB1, and MAPK1 with erlotinib treatment were not correlated with their respective transcripts, which may suggest that the change in phosphorylation was due to altered signaling and not due to transcription and translation. Tissue IF data provided the bridge between response in flank and IC tumors and showed that EGFR and p-EGFR were not inhibited at the limited IC drug exposure.

Analysis of mRNA isolated from four quadrants of the high-dose IC tumor, suggested that the IC-treated tumors were more similar to these two lower dose flank cohorts at the transcript level. While the differential gene expression and ingenuity pathway for the IC quadrants was limited from having taken all genes into account (filtering out low abundance genes and batch sequence effect), the overall result provided evidence that similar dose-related response was observed in IC and flank tumors exposed to relatively similar drug levels. It should be noted that IC tumors were analyzed following a single dose of erlotinib (2 h post dose), whereas flank tumors were dosed three times over 3 days and sacrificed 6 h post final dose. After two hours, the concentration of drug in IC tumors is similar to those measured in low dose flank (by MALDI MSI) and transcriptomic analysis of mRNA signatures validates the drug concentration. In addition, mRNA transcripts change quickly (within 1–2 h) which is consistent with the IC tumor transcript signature being similar to that from flank tumor at low dose.

Considering the wider implications of this study it is important to account for model specific limitations. The integrity of the BBB in the mouse is clearly a critical factor for assessment of PK; a recent comparison of BBB permeability in the athymic nude mouse and an immunocompetent mouse found no difference in drug (ponatinib, another tyrosine kinase inhibitor) concentration in plasma and normal brain tissue^34^. Compromise of the BBB in brain tumors has been shown to be largely ascribed to tumor cell secretion of cytokines which leads to localized effects on tight junctions, as well as other structural features of the BBB^35^. This cytokine effect on the BBB has shown to be present in athymic mice^35^ suggesting that the PDX model described herein is a suitable model for PK/PD investigations in GBM. The use of PDX models requires a deficient immune system and therefore the inflammatory response will be compromised; in future work we will consider the use of genetically engineered mouse models of GBM in order to retain and study the effect of an intact immune system. In addition to the possible issues surrounding the use of PDX models; in this work we considered a single GBM xenograft model, it is likely that due to heterogeneity in genotype and phenotype between different GBM tumors, differences in PK and PD would be observed in other models.

Overall we propose that significant portions of IC PDX tumors received insufficient drug levels to successfully inhibit EGFR tyrosine kinase signaling, and that this might be due to both limitations imposed by the BBB and tissue characteristics. This conclusion was drawn based on imaging the distribution of erlotinib in relation to other chemical and functional markers, and demonstrates the power of the multimodal platform in establishing relationships between drug PK and PD.

## Methods

### Animals

All studies involving animals were reviewed and approved by the Institutional Animal Care and Use Committee at Mayo Clinic. Xenografts were established in female athymic nude mice (Hsd:athymic Nude-Foxn1^nu^, ages 6–7 weeks; Envigo, Indianapolis, IN) as previously described^36,37^. The GBM12 PDX model has been previously described^38^, full genotypic and phenotypic characterization is available from the Mayo Clinic PDX National Resource (http://www.mayo.edu/research/labs/translational-neuro-oncology/mayo-clinic-brain-tumor-patient-derived-xenograft-national-resource). A study into GBM12 IC PDX model survival with erlotinib treatment demonstrated a modest improvement in overall survival in mice treated with either 100 or 150 mg kg^−1^ erlotinib daily for two weeks or until moribund, compared with a control group, demonstrating the suitability of this model/drug combination for the study described herein. The provenance of the PDX models used have been confirmed by short tandem repeat analysis with a comparison to the original patient tumor or germline DNA sample. All of the PDX models are maintained by serial heterotopic tumor passage at Mayo Clinic. Previous testing of these PDX models for Mycoplasma has uniformly been negative. Mice were stratified by tumor size and randomized to treatment groups using the StudyLog database program (http://www.studylog.com/). Mice with established IC or flank tumors were dosed with a single dose of placebo, 33 or 100 mg kg^−1^ erlotinib. Two hours post dose mice were euthanized by CO_2_ inhalation and brain and plasma were harvested and flash frozen on dry ice for multi-modality analysis.

Adaptation phosphoproteomics and mRNA sequencing experiments were performed on a further set of mice with established flank tumors and were treated when tumor volume reached 250–350 mm^3^. Treatments were placebo, 5, 33, or 100 mg kg^−1^ erlotinib daily for 3 days (total three doses) and 6 h after last dose mice were euthanized with CO_2_ inhalation and tumor tissue and plasma were harvested and flash frozen on dry ice for subsequent analysis. This study included five mice per treatment group, the selection of animal number was based on the feasibility of the downstream analyses (proteomics, RNAseq, MALDI) across multiple treatment groups. The number of mice used for each analysis is stated for each method separately. All mice were observed daily by a veterinarian blinded to the treatment received. The technician performing the tumor measurements was also blinded to the treatment received.

### Sample preparation for MALDI MSI

Brain or flank tumors were dissected and snap frozen by immersion in liquid nitrogen. Tissues were placed at −25 °C 1 h before use, and tissue sections were prepared using a Microm HM550 cryostat (Thermo Scientific™, Waltham, MA) with the microtome chamber chilled at −20 °C and the specimen holder at −19 °C. Cryosections of 12-μm thickness were thaw mounted onto ITO-coated microscopic slides (Bruker Daltonics, Billerica, MA) for MALDI MSI and onto optical slides for hematoxylin, eosin (H/E) staining, and Tissue IF. For MALDI MSI, the mounted sections were dried for 15 min in a desiccator prior to matrix application. 2,5-dihydroxybenzoic acid matrix (DHB, 160 mg mL^−1^ solution in methanol/0.2% TFA 70:30 vol/vol) was deposited using a TM-sprayer (HTX imaging, Carrboro, NC) with the following conditions: flow rate, 90 μL min^−1^; spray nozzle velocity, 1200 mm min^−1^; spray nozzle temperature, 75 °C; nitrogen gas pressure, 10 psi; track spacing, 2 mm; number of passes, 4.

### Tissue mimetic sample preparation for quantitative MALDI

100 μL aliquots of control mouse brain tissue homogenate were transferred into 7 pre-weighed 1.5 mL Eppendorf tubes and then each tube was reweighed to provide an accurate mass for total quantity of homogenate transferred. Based on the weight of the tissue homogenate in each tube the appropriate concentration of drug standard was added at concentrations of 50,000, 10,000, 7500, 5000, 2500, 1000, 500 ng g^−1^ (drug/homogenate). Based on the work of Groseclose et al.^29^, we developed a 3D model of their negative mold and printed it using a 3D printer. The negative mold was filled with 40% gelatin and placed at −80 °C for 2 h. Once frozen, the assembly was placed into the chamber of a cryostat held at −40 °C, the negative mold was removed, and the frozen cylinder of 40% gelatin was separated from the outer tube by removing the cap and sliding out the 40% gelatin. The 100 μL-spiked tissue homogenates were then transferred into separate cores in the frozen cylinder of 40% gelatin inside the cryostat chamber. The tissue model was placed at −80 °C for 24 h prior to sectioning. The tissue mimetic model was then sectioned at 12-µm thickness using a Microm HM550 cryostat and sections were thaw mounted onto ITO-coated microscopic slides next to the tissue sections for MSI.

### MR imaging

Magnetic resonance imaging (MRI) was performed using a Bruker DRX-300 (300 MHz 1 H) 7 T vertical-bore small animal imaging system (Bruker Biospin, Billerica, MA) similarly to published protocols.^39^ Throughout imaging, mice were anesthetized by inhalation of 3–4% isofluorane in air and their respiratory rate monitored. For T1 weighted imaging, mice were administered Gd contast agent (Gadavist^®^) intraperitoneally (i.p.) at a dose of 100 mg kg^−1^ and imaged after a 15 minute delay. T2 weighted MRI: RARE pulse sequence, repetition time (TR) = 1500 ms, echo time (TE) = 70 ms, RARE factor: 16, field of view (FOV): 3.2 × 1.92 × 1.92 cm, matrix: 256 × 128 × 128. T1 weighted MRI: MSME, TR: 300 ms, TE: 9.5 ms, FOV: 4.0 × 2.0 × 2.0 cm, matrix: 192 × 96 × 96.

### MALDI mass spectrometry imaging

Mass spectra were acquired using a 9.4 T SolariX XR FT-ICR MS (Bruker Daltonics, Billerica, MA) externally calibrated in electrospray ionization positive ion mode using a tuning mix solution (Agilent Technologies, Santa Clara, CA). MALDI MS images were acquired with a pixel step size for the surface raster set 100 µm for 3D imaging of brain sections and 175 µm for quantitative MALDI MS imaging. Spectra were acquired in positive ion mode with 250 laser shots accumulated at each location with a range of *m/z* 380–620 for erlotinib/M13/M14 metabolites/heme. The laser intensity was set to 15% intensity to desorb/ionize the drug with a laser frequency of 1000 Hz. MALDI MS images were displayed and analyzed using FlexImaging 4.0 and SCILS software.

### Liquid chromatography–tandem mass spectrometry

Erlotinib hydrochloride (purity > 99%) was purchased from LC Laboratories (Woburn, MA). [^13^C_6_]-erlotinib hydrochloride (purity > 98%) was purchased from Alsachim SAS (Illkirch, France). Analytical-grade reagents were purchased from Thermo Fisher Scientific (Waltham, MA). The erlotinib concentrations in plasma, flank tumor, and brain resulting from in vivo animal experiments are measured using an reverse-phase liquid chromatography (Agilent model 1200 separation system; Agilent Technologies, Santa Clara, CA) interfaced with TSQ Quantum triple quadrupole mass spectrometer (Thermo Finnigan, San Jose, CA) by operating electrospray in positive ion mode and spray voltage at 28 V. Brain and flank tumor samples were homogenized with three tissue volumes of 5% bovine serum albumin (g/v) solution using a mechanical homogenizer (PowerGen 125; Thermo Fisher Scientific, Waltham, MA). Liquid–liquid extraction was performed by adding 75 ng of [^13^C_6_]-erlotinib hydrochloride (internal standard), 25-μL aliquot of plasma or 50-μL aliquot of tissue homogenate, ten volumes of ice-cold ethyl acetate, and two volumes of pH 11 buffer (1 mM sodium hydroxide, 0.5 mM sodium bicarbonate). Samples were vortexed for 5 min, centrifuged at 7500 rpm for 5 min (4 ^o^C), and the organic layer was dried under nitrogen. The dried drug was reconstituted with 150 µL of mobile phase (water with 0.1% formic acid:acetonitrile with 0.1% formic acid = 50:50), followed by centrifugation at 14,000 rpm for 5 min (4 ^o^C). 2 µL of the sample was injected into the Phenomenex Synergi 4 µm polar-RP 80 Å LC column (75 × 2 mm; Torrance, CA) for chromatographic separation. The mobile phase was delivered at a flow rate of 0.25 mL min^−1^. The retention time was 1.7 min for erlotinib hydrochloride and [^13^C_6_]-erlotinib hydrochloride. The total run time was 5 min. The mass-to-charge ratio (*m*/*z*) transitions were 394.44 → 278.00 for erlotinib hydrochloride and 400.20 → 284.10 for [^13^C_6_]-erlotinib hydrochloride. The calibration curve was linear and precise over a range of 1–3000 ng mL^−1^ (weighting factor of 1/Y^2^) with the coefficient of variation of <15%^26^.

### SRS microscopy

The principle and setup of label-free SRS microscope have been previously described^40^. Briefly, the SRS microscope was composed of a dual-color narrow-band laser source (picoEmerald, APE), providing the tunable pump beam (720–990 nm, pulse width 5–6 ps) and the Stokes beam at a fixed wavelength (1064 nm, pulse width, 7 ps), and an upright laser-scanning confocal microscope (FV300, Olympus). The pump and Stokes beams were collinearly combined with a dichroic mirror, introduced into the microscope via the two-dimensional scanning head, and then were tightly focused onto the sample using a water-immersion objective (XL PLAN N 25 ×, NA 1.05; Olympus). Imaging was realized by raster scanning the laser beams across the sample. Frequency difference between the two beams determines the Raman shifts to image. By tuning the pump wavelength, multicolor imaging could be achieved. The laser source was remote controlled through the RS-232 interface. A single FOV was 350 × 350 μm. Each FOV was acquired with 512 × 512 pixels in about one second. Tiling imaging was conducted using an automated stage (MS2000, ASI) with partial overlapping. To realize high-sensitive SRS imaging free from the laser noise, the Stokes beam was amplitude-modulated at 10 MHz using an electro-optic modulator (EOM, Thorlabs), and the modulation transferred to the pump beam (i.e., stimulated Raman loss) was detected using a home-built all-analog lock-in amplifier^41^. A Si photodiode detector was used to convert the pump light to electrical signals for detection. Labview programming (National Instruments Inc.) integrated the whole system for automatic multi-color and large-tissue image acquisition. Lipids were imaged (green) at 2854 cm^−1^ attributed to CH_2_ stretching vibration from the fatty acid long chains; proteins were imaged (blue) at 2940 cm^−1^ mainly attributed to CH_3_ vibration of the chemical bonds in the amino acids; heme (red) was imaged at 2800 cm^−1^ due to two-color two-photon absorption. Imaging resolution is ~500 nm. Scale bar, 350 μm.

### Tissue IF staining and microscopy

Localization of total and phosphorylated (Tyr1173) EGFR in tumor sections from flank and brain tumors was examined via IF staining. Briefly, 8 μm tumor sections were fixed on glass slides in 4% paraformaldehyde for 10 min at room temperature (RT) and rinsed with PBS. Then, the cells were permeabilized in PBS containing 0.1% Triton X-100 for 3 min. After washing with PBS, blocking buffer (5% goat serum/TBS) was applied to block unspecific staining for 1 h at RT. The samples were incubated with primary antibody (anti-EGFR 1:200 (ab32077) or anti-phospho-EGFR 1:50 (ab32578)) in 5% goat serum/TBS-0.05% Tween at 4 °C overnight, and subsequently the secondary antibody (Invitrogen, A11036) was conjugated with Alexa Fluor^®^ 568 and nuclei were stained with Hoechst. A negative control leaving out the primary antibody was included. Fluorescence images were acquired using a Zeiss Observer Z.1 microscope equipped with ×20 Plan-APOCHROMAT lens and AxioCam MR3 camera. Fluorescence images of Hoechst and EGFR/p-EGFR were acquired using a fluorescence light source (X-Cite Series 120Q) and excitation/emission wavelengths at 365/445 and 554/581 nm, respectively. Exposure times were optimized visually and fixed at 329 µs for EFGR and p-EGFR, and 24 µs for Hoechst. Images were processed using Zen Pro 2012 (Blue Edition) and ImageJ. Images of the entire tissue section (brain or flank tumor) were acquired and subsequently ROI were created manually using Hoechst images to segment the tumor region in brain and the full flank tumor. ROIs were transferred to EGFR/p-EGFR and average intensity over the whole tumor region was calculated (*n* = 3 per treatment group flank tumors, *n* = 2 per treatment group IC tumors). Statistical significance was tested using an unpaired, two-tailed *t*-test. The species reactivity for anti-p-EGFR is human (http://www.abcam.com/egfr-phospho-y1173-antibody-e124-ab32578.html) and for anti-total-EGFR is rat, mouse, and human (http://www.abcam.com/egfr-antibody-e235-ab32077.html).

### Automatic multi-modal non-rigid image registration

The MALDI MSI datacube was non-linearly aligned to both H&E histology and MRI using the automatic t-distributed stochastic neighbor embedding (t-SNE) based registration method^42^. Briefly, the dimensionality reduction method of the t-SNE^43^ was first used to project the hyper-dimensional MALDI MSI datacube into a 3D space for which coordinates represent t-SNE features. Those 3D t-SNE features were spatially organized as such each t-SNE feature represents an image that was used to feed a color channel in a L*a*b* color system, and the resultant combined image is called t-SNE image. Of note, the spectra were projected while preserving their local characteristics, in a single map representation in 3D space, based on their pairwise similarities in which similar spectra were projected close to each other whereas dissimilar ones were projected far apart. This translates to forming molecularly distinct structures in the t-SNE image and thus establish spatial correspondences that would guide the registration with other structure/anatomy-rich images, such as histology and MRI.

The t-SNE image was then non-linearly aligned to its corresponding 2D MR image. A 2D MR image was manually selected from the MRI volume at similar location to that of a MALDI section. In this alignment process, the t-SNE image was first linearly warped using Affine transformation (T_A) to capture global differences (translation, rotation, scaling, and shearing), and then non-linearly warped using the BSpline transformation (T_B) to correct for local differences. And finally, each *m/z* image was transformed using T_A and T_B, respectively, to be spatially aligned with the MR image. This alignment process was repeated but after replacing the MR image with the corresponding histological image. The alignment quality was visually assessed.

### Phosphoproteomic analysis

Tumor material (whole flank tumor) was collected from mice (*n* = 4 per treatment group: vehicle, 5 mg kg^−1^ erlotinib, 33 mg kg^−1^ erlotinib, and 100 mg kg^−1^ erlotinib) and snap frozen in liquid nitrogen. Tumors were homogenized by sonication in 8 M urea supplemented with 1 mM sodium orthovanadate (Na_3_VO_4_), 0.1% Nonidet P-40, protease inhibitor cocktail (Roche) (1 tablet/10 mL), and PhosStop (Roche) (1 tablet/10 mL). Protein concentration was measured by a bicinchoninic acid (BCA) assay (Pierce) and proteins were reduced with 10 mM dithiothreitol (DTT) for 1 h at 56 °C, alkylated with 50 mM iodoacetamide for 1 h at RT, and diluted fourfold with 100 mM ammonium acetate at pH 8.9. Proteins were digested using trypsin (sequencing-grade, Promega; 1 μg trypsin:50 μg total protein) overnight at RT. Enzyme activity was quenched by acidification of the samples with acetic acid. The peptide mixture was desalted and concentrated on a C18 Sep-Pak Plus cartridge (Waters) and eluted with 40% acetonitrile, 0.1% acetic acid. Organic solvent was evaporated in a SpeedVac vacuum centrifuge. 400 μg aliquots of each sample were aliquoted and frozen in liquid nitrogen for 5 min, lyophilized and stored at −80 °C. Tumors were processed and labeled with tandem-mass tag (TMT)−10-plex Mass Tag Labeling Kits (Thermo)^44^. One vehicle-treated and one erlotinib-treated (5 mg kg^−1^, 33 mg kg^−1^, 100 mg kg^−1^) tumor was included in each of a total of two TMT-10-plex analyses. Thus, allowing them to serve as normalization channel, as well as technical replicates. For TMT labeling, tumor peptide aliquots (400 μg peptide for each channel) were resuspended in 100 μL of 70% (vol/vol) ethanol, 30% (vol/vol) 0.5 M triethylammoniumbicarbonate at pH 8.5, and incubated with TMT reagent resuspended in 40 μL anhydrous acetonitrile at RT for 1 h. The samples were concentrated, combined, and concentrated to dryness using a SpeedVac centrifuge. Phosphopeptides were enriched using phosphotyrosine immunoprecipitation and immobilized metal ion chromatography (IMAC)^44^. In brief, dried samples were resuspended in IP buffer (100 mM Tris–HCl, 1% Nonidet P-40 at pH 7.4) and incubated with protein G agarose beads conjugated with 12 μg of anti-phospho tyrosine antibodies: 4G10 (Millipore), PY-100 (Cell Signaling Technologies), and PT-66 (Sigma) overnight at 4 °C. Due to differences in sequence in preferences of phosphotyrosine-targeting antibodies^45^, we combined three different antibodies to maximize the sequence coverage of different phosphorylated tyrosine-containing peptides by the immunoprecipitation. Beads were washed with IP buffer and rinse buffer (100 mM Tris–HCl at pH 7.4) and subsequently peptides were eluted with 100 mM glycine at pH 2.5 for 30 min at RT and acidified with TFA. Next, phosphopeptides were additionally enriched by IMAC using Fe-NTA Agarose beads. Bound peptides were eluted with 250 mM NaH_2_PO_4_ and loaded onto a precolumn [100 μm ID × 10 cm packed with 10 μm C18 beads (YMC gel, ODS-A, 12 nm, S-10 μm, AA12S11)], which was rinsed with 0.2 M acetic acid for 10 min before LC–MS analysis^44^. Raw mass spectral data files were loaded into Proteome Discoverer version 1.4.1.14 (DBversion: 79) (Thermo) and searched against the human SwissProt database (including 20,215 sequences) using Mascot version 2.4 (Matrix Science). TMT reporter quantification was extracted and isotope corrected in Discoverer. Tandem mass spectra were matched with an initial mass tolerance of 10 ppm on precursor masses and 15 mmu for fragment ions. Cysteine carbamidomethylation, TMT-labeled lysine, and protein N-terminal were searched as fixed modifications. Oxidized methionine, and phosphorylation of serine, threonine, and tyrosine were searched as variable modifications. Minimal peptide length was seven amino acids. Only peptides with an ion score > 20 were included in the final peptide list. Peptide quantification was normalized based on median relative protein quantification obtained from a crude peptide analysis to correct for minor variation in sample amount among TMT channels. For each peptide, relative quantification was represented as a ratio between TMT ion intensities for each analyzed tumor and the included normalization channel. The protein network was obtained by using the STRING database (version 10.0)^46^. All active interaction sources except text mining were included and to ensure high confidence, a confidence score over 0.9 was required. Further network analysis and visualization were performed by using the Cytoscape platform (version 2.8)^47^.

### RNA isolation and sequencing

ArcturusXT™ LCM System was used to capture cells from four regions of the IC tumor section. Total RNA from each captured region were isolated using The ARCTURUS^®^ PicoPure^®^ RNA Isolation kit according to manufacturer’s protocol. For flank tumors, total RNA was isolated using RNAeasy Mini Kit (Qiagen) according to manufacturer’s protocol.

Total RNA (50 ng) for each region was used to generate whole transcriptome libraries for RNA sequencing using Illumina’s TruSeq RNA Sample Prep Kit (catalog #FC-122-1001) following the manufacturer’s recommendation. Poly-A mRNA selection was performed using oligo(dT) magnetic beads, and libraries were enriched using the TruSeq PCR Master Mix and primer cocktail. Amplified products were cleaned and quantified using the Agilent Bioanalyzer and Invitrogen Qubit. The clustered flowcell was sequenced on the Illumina HiSeq 2500 for paired 100-bp reads using Illumina’s TruSeq SBS Kit V3 (catalog #FC-401-3001).

### Transcriptome alignment and differential expression analysis

Lane level fastq files were appended together if they were sequenced across multiple lanes. These fastq files were then aligned with STAR 2.3.1 to GRCh37.62 using ensembl.63.genes.gtf as GTF file. Changes in transcript expression were calculated with Cuffdiff 2.1.1 in FPKM format using upper-quartile normalization. Clustering was performed using R heatmap.2 package with Euclidean Distance and McQuitty clustering method. Files submitted to the European Nucleotide Archive (ENA).

### Clustering of phosphoproteomic and transcriptomic data

All 212 phosphopeptides detected by mass spectrometry and the subset of 5002 transcripts that are differentially expressed either in the placebo condition vs. any treatment condition, or in any of the four control and treatment conditions vs. the other three were analyzed. Replicate spectrometric measurements from the same tumor were averaged geometrically before use. A pseudocount of 0.001 FPKM was added to each transcriptomic measurement, and then the data were normalized to control tumor 2786 and log-transformed. Pearson correlation coefficients were calculated for each phosphopeptide and transcript with each other across tumor samples. Vectors of correlation coefficients within or across classes of measurement were clustered hierarchically using the Euclidean distance metric, average linkage, and leaf ordering to maximize the similarity between neighboring leaves using Matlab version R2017b.

## Electronic supplementary material

Supplementary Information

Description of Additional Supplementary Files

Supplementary Data 1

Supplementary Data 2

Supplementary Data 3

## Data Availability

The mass spectrometry proteomics data have been deposited to the ProteomeXchange Consortium via the PRIDE partner repository with the dataset identifier PXD006769. Binary sequence alignment/map (BAM) files of RNA-seq data from GBM12 flank and intracranial studies are available from the EMBL-EBI European Nucleotide Archive (ENA) database - http://www.ebi.ac.uk/ena/ with accession number PRJEB25377. For the intracranial study, the sample accession numbers are ERS2269279–ERS22692780 and ERS2272583–ERS2272584. For the flank study, the sample accession numbers are ERS2280157–ERS2280161 and ERS2310063–ERS2310074. All other data is available upon request.
