# Supplementary material for: Integrated mapping of pharmacokinetics and pharmacodynamics in a patient-derived xenograft model of glioblastoma
Source: Nat Commun. 2018 Nov 21;9:4904. doi: 10.1038/s41467-018-07334-3 (PMC6249307; doi:10.1038/s41467-018-07334-3)
Supplement: Supplementary file 1 — Supplementary Information [file 41467_2018_7334_MOESM1_ESM.pdf]

Integrated mapping of pharmacokinetics and pharmacodynamics in a patient-derived xenograft model of glioblastoma

Elizabeth C. Randall[a], Kristina B. Emdal[b], Janice K. Laramy[c], Minjee Kim[c], Alison Roos[d], David Calligaris[e], Michael S. Regan[e], Shiv K. Gupta[f], Ann C. Mladek[f], Brett L. Carlson[f], Aaron J. Johnson[g], Fa-Ke Lu[e,h,i], X. Sunney Xie[h], Brian A. Joughin[b], Raven J. Reddy[b], Sen Peng[j], Walid M. Abdelmoula[e], Pamela R. Jackson[k], Aarti Kolluri[k], Katherine A. Kellersberger[l], Jeffrey N. Agar[m], Douglas A. Lauffenburger[b], Kristin R. Swanson[k], Nhan L. Tran[d], William F. Elmquist[c], Forest M. White[b], Jann N. Sarkaria[f], and Nathalie Y. R. Agar[a,e,n]

Supplementary Information

Supplementary Figures 1 – 8

Supplementary Tables 1 – 2

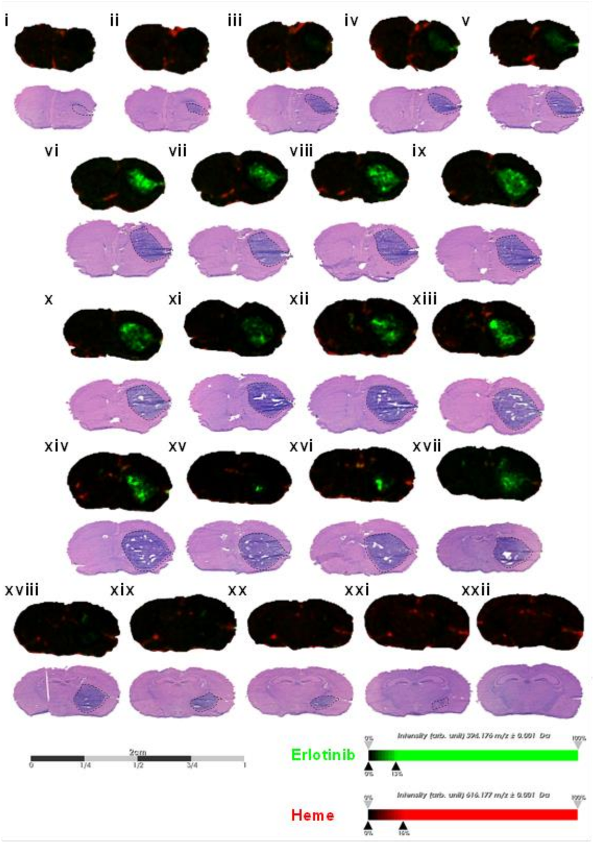

Sample	m/z exp	m/z th	Mass Accuracy (ppm)
VI	394.1758	394.1761	0.761081731
VII	394.1758	394.1761	0.761081731
VIII	394.1758	394.1761	0.761081731
IX	394.1758	394.1761	0.761081731
X	394.1758	394.1761	0.761081731
XI	394.1760	394.1761	0.253693782
XII	394.1760	394.1761	0.253693782
XIII	394.1762	394.1761	-0.253693653
XIV	394.1760	394.1761	0.253693782
XV	394.1763	394.1761	-0.507387177
XVI	394.1764	394.1761	-0.761080572
XVII	394.1758	394.1761	0.761081731

Supplementary Fig. 1 Measured and theoretical *m/z* of erlotinib detected from each tissue section and corresponding Δppm.

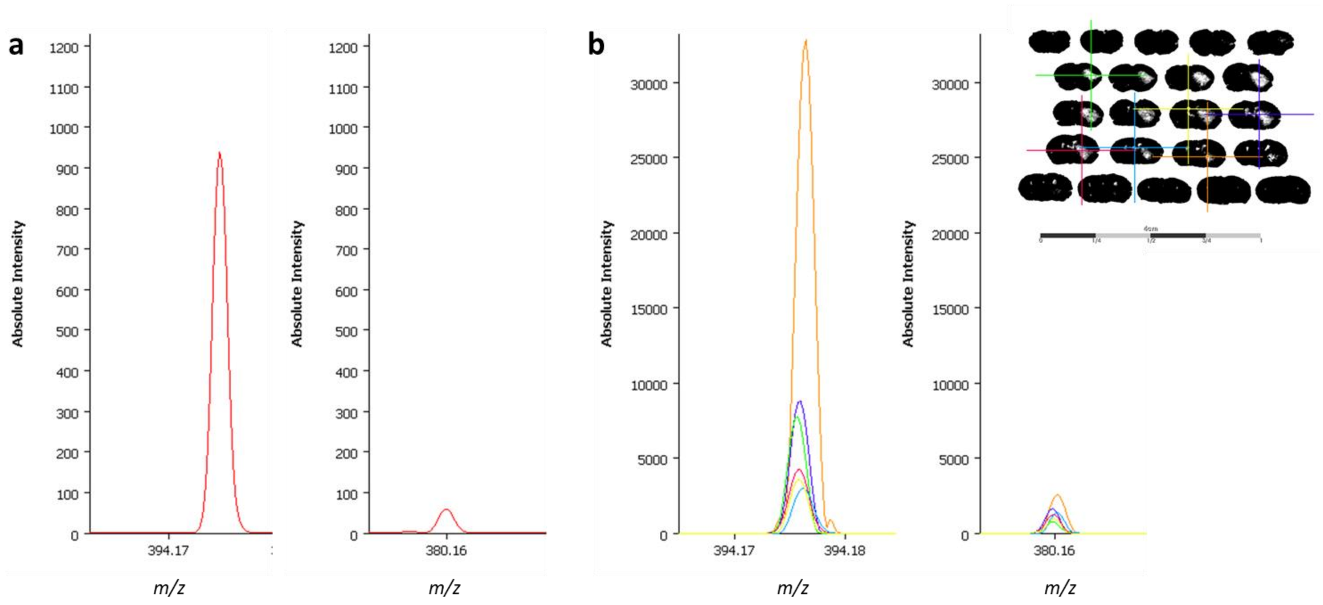

Supplementary Fig. 2 Comparison of relative peak intensities detected at *m/z* 394.1760 and 380.1760 corresponding to ions of erlotinib and erlotinib metabolite M13/M14 respectively, a) average mass spectrum over all regions analyzed and b) example single pixel spectra from locations indicated inset.

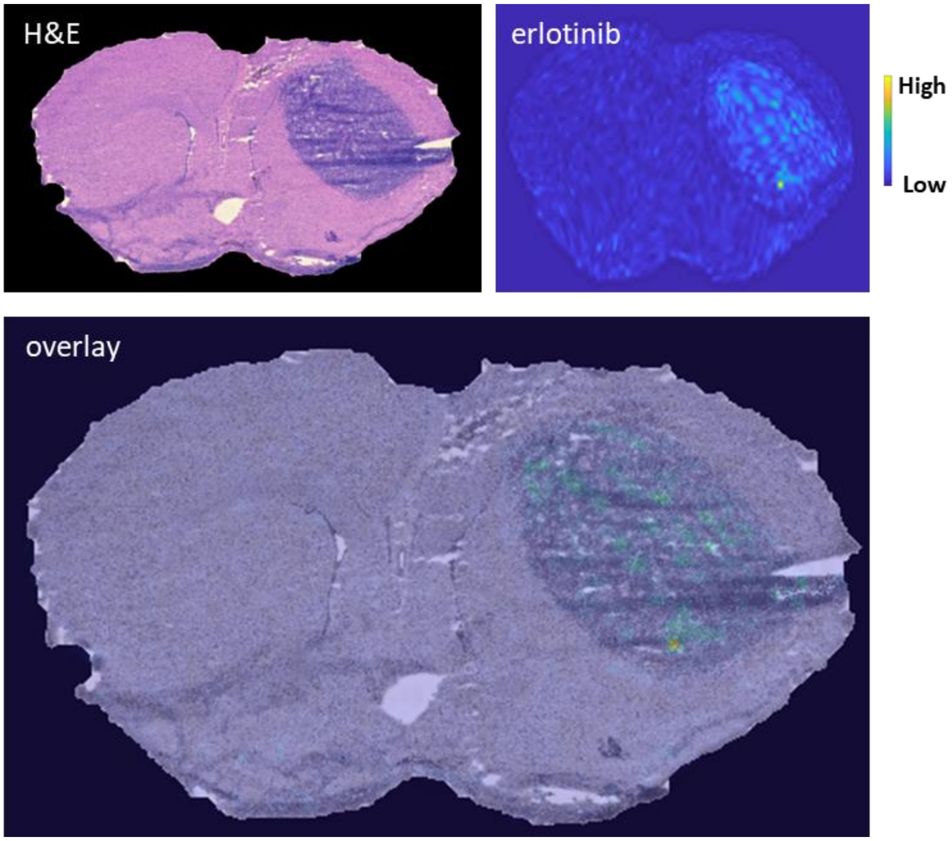

Supplementary Fig. 3 Registration of H&E image and MALDI MS ion image of erlotinib.

a

b

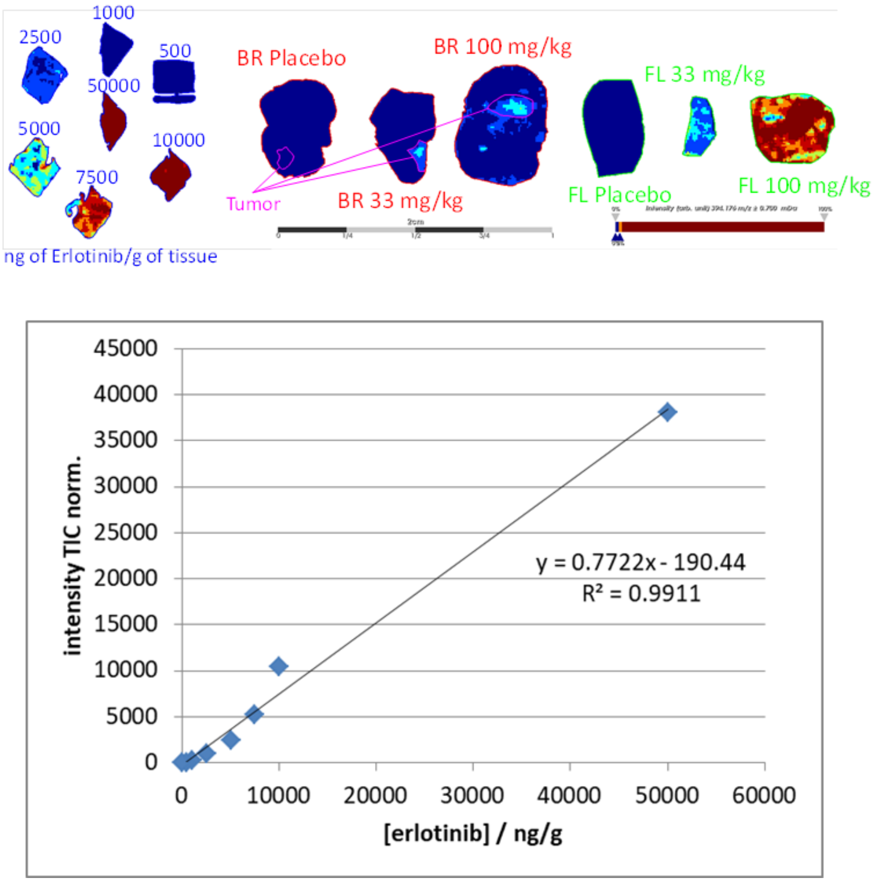

Supplementary Fig. 4 Erlotinib quantification from MALDI MSI of mouse brain (BR) sections with GBM tumors and GBM flank (FL) tumor sections at 2-hour dosing time (placebo or dosage 33mg/kg, and 100 mg/kg). a) Ion image of Erlotinib (*m/z* 394.176 ± 0.001) displaying the relative abundance of the drug within the tissue mimetic model, brain, and flank sections. Sections were imaged at 125 µm spatial resolution by MALDI MSI. b) Data series in blue indicate the calibration curve constructed by plotting the maximum intensity values for the monoisotopic Erlotinib peak in the average mass spectra of each homogenate core of the tissue mimetic model (500 − 50000 ng/g). The red, green, and pink data points display the maximum intensity values from the monoisotopic Erlotinib peak in the average mass spectra of the brain, and flank sections (delineated by red and green lines on the MALDI MSI image), and the tumor area within the brain sections (delineated by a pink line on the MALDI MSI image), respectively.

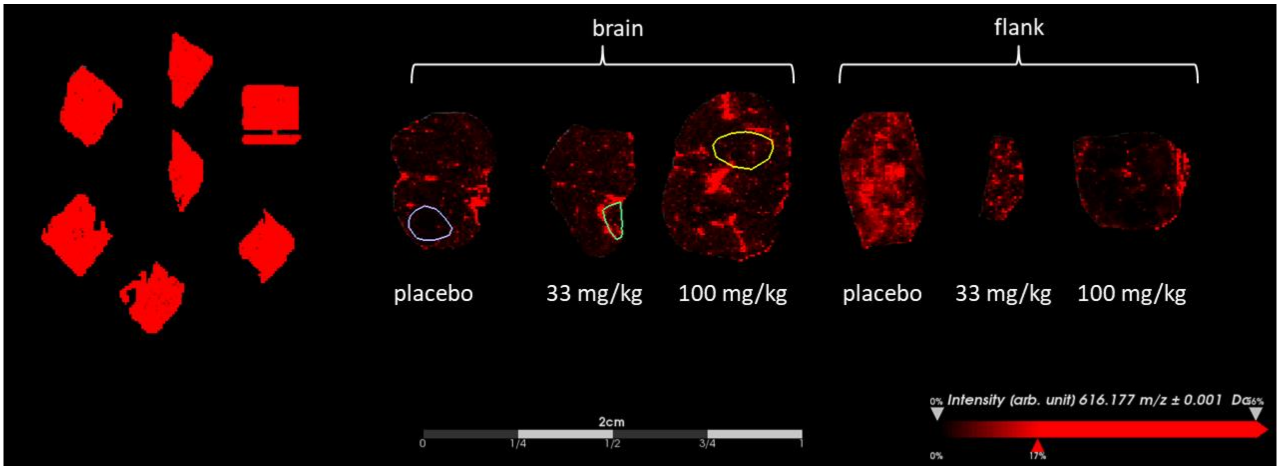

Supplementary Fig. 5 MALDI ion image of ions with m/z 616.176 (heme) in the dataset used for quantitative measurement of erlotinib, high intensity of heme can be seen around and within tumor region for 33 mg/kg intracranial tumor. Regions of interest delineating IC tumor from normal brain are displayed inset.

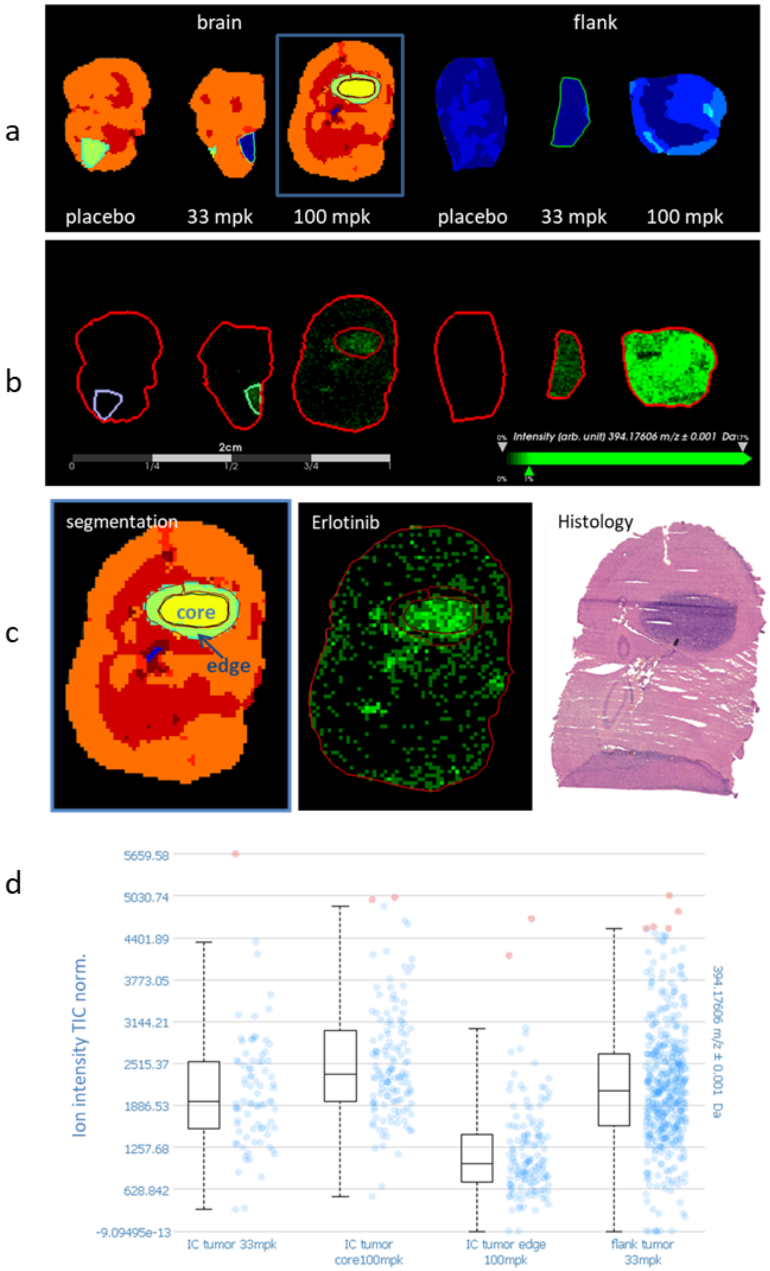

Supplementary Fig. 6 Segmentation of MALDI MSI data for calculation of concentration of erlotinib in IC PDX, a) bisecting *k*-means clustering of full MSI dataset (k = 16), ROIs indicate IC tumor region, manually selected from segmentation map, b) MALDI MS ion image of ions with *m/z* 394.176 (erlotinib), c) enlarged view of segmentation map for brain dosed with 100 mg/kg erlotinib, with ROI delineating tumor core (yellow) and tumor edge (green), with associated erlotinib ion image and histology of serial section, d) boxplot of ion intensity for *m/z* 394.176 over different doses in IC and flank tumors and segmented regions of IC tumor, alongside scatterplot of individual datapoints.

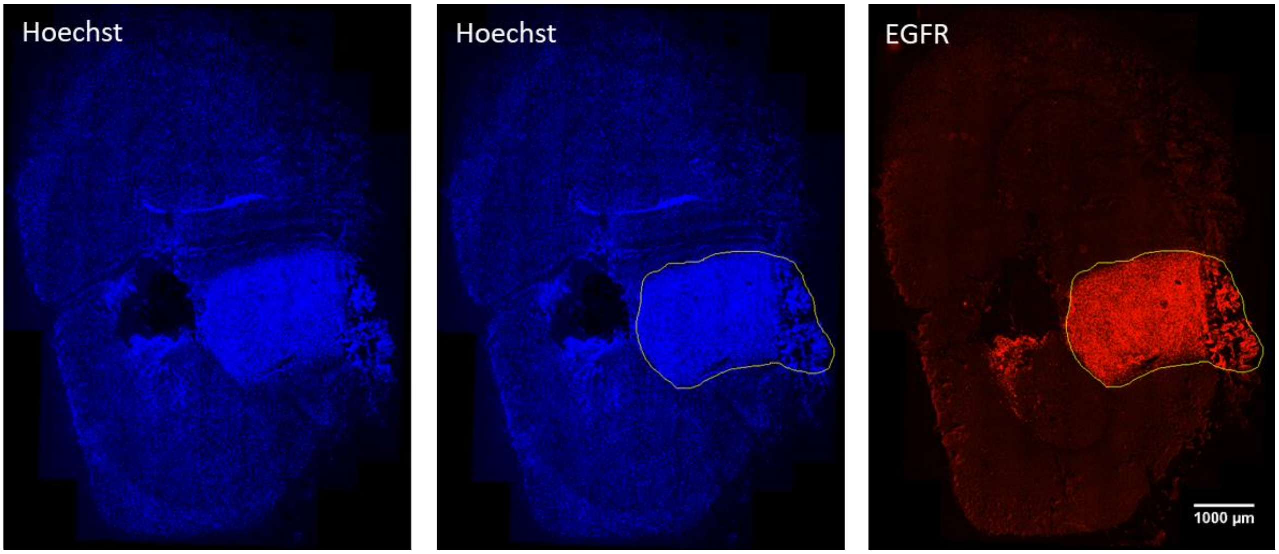

Supplementary Fig. 7 Example images showing description of image analysis for tissue IF, ROIs were manually drawn around tumors in Hoechst image using ImageJ software (shown in yellow), this ROI was then copied to either the EGFR or phosphorylated-EGFR (not shown) image and average intensity within tumor ROI was calculated. EGFR is detected with higher intensity from the tumor (outlined in yellow) compared with normal brain tissue (in background).

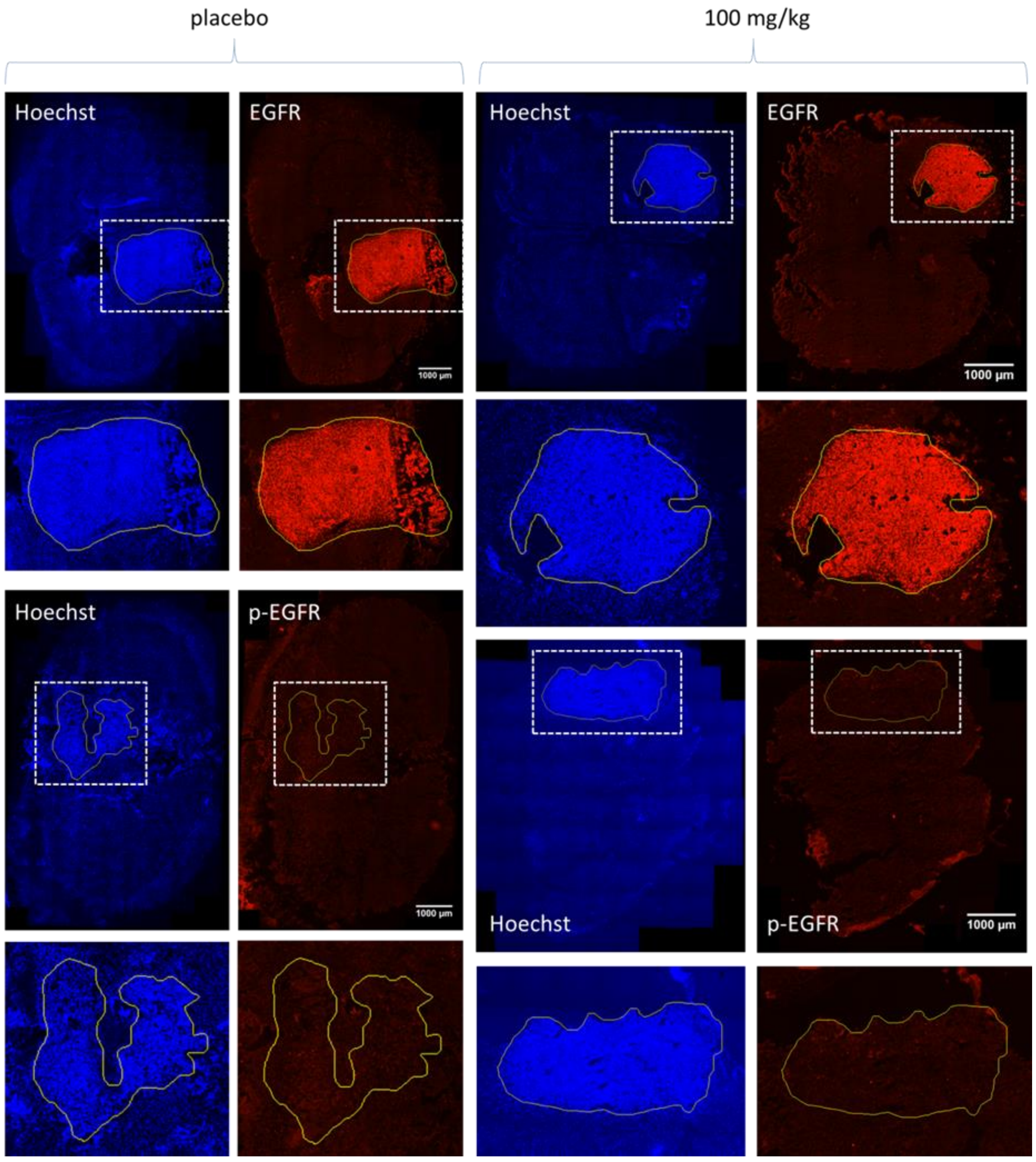

Supplementary Fig. 8 Example fluorescence images (Hoechst in blue, EGFR/p-EGFR in red) of IC PDX tumors from placebo and 100 mg/kg treatment groups, selections displayed in yellow indicate manual segmentation of tumors for quantitative assessment. Variable intensity of EGFR and p-EGFR was observed throughout tumor regions under all treatment conditions. Scale bar = 1000 µm

Supplementary Table 1 correlation matrix of Pearson's correlation coefficient for selected ion images corresponding to erlotinib (*m/z* 394.176), M13/M14 metabolite (*m/z* 380.160), heme (*m/z* 616.177) and a tumor biomarker (*m/z* 503.949).

		Ion image			
		**tumor marker**	**m13/m14**	**erlotinib**	**heme**
**Ion image**	**tumor biomarker**	1	0.227	0.294	0.00377
	**m13/m14**	0.227	1	0.628	0.014
	**erlotinib**	0.294	0.628	1	0.0123
	**heme**	0.00377	0.014	0.0123	1

Supplementary Table 2 Correlation coefficient between heme and drug ion images from MALDI MSI data, higher correlation was observed between heme and drug in IC tumors compared with flank, suggesting the drug is less available in IC tumors.

ROI from MALDI MS image	calculated mean concentration per region ng/g	correlation coefficient drug/heme
flank tumor 100 mg/kg	8211.6	0.0090
flank tumor 33 mg/kg	2426.7	0.0098
flank tumor placebo	<LOD	n/a
IC tumor 100 mg/kg	2182.3	0.0268
IC tumor 33 mg/kg	2354.2	0.0199
IC tumor core100 mg/kg	2787.1	0.0146
IC tumor edge 100 mg/kg	1446.5	0.0415
IC tumor core 33 mg/kg	2411.5	0.0099
IC tumor edge 33 mg/kg	2011.2	0.0192
IC normal hemisphere 100 mg/kg	1082.9	0.027
IC normal hemisphere 33 mg/kg	619.3	9.45E-05
IC normal hemisphere placebo	<LOD	n/a
