# Supplementary material for: Integrated mapping of pharmacokinetics and pharmacodynamics in a patient-derived xenograft model of glioblastoma
Source: Nat Commun. 2018 Nov 21;9:4904. doi: 10.1038/s41467-018-07334-3 (PMC6249307; doi:10.1038/s41467-018-07334-3)
Supplement: Supplementary file 2 — Description of Additional Supplementary Files [file 41467_2018_7334_MOESM2_ESM.docx]

**Description of Additional Supplementary Files**

File Name: Supplementary Data 1

Description: Phosphoproteomic analysis of flank tumors: individual tumors treated with 33 or 100 mg/kg erlotinib demonstrated increased phosphorylation of selected nodes, potentially indicative of resistance pathways beginning to emerge in these tumors.

File Name: Supplementary Data 2

Description: Correlation matrix of 210 tyrosine phosphorylation sites across flank tumors under placebo, ultra-low, low and high doses of erlotinib.

File Name: Supplementary Data 3

Description: mRNAseq data filtered for those that are differentially expressed either between any single erlotinib condition and the DMSO control, or between any one condition and the other three (as a pool).
